# Different responses of larval fatty acid profiles to cryopreservation in two commercially important bivalves

**DOI:** 10.1038/s41598-024-76723-0

**Published:** 2024-10-19

**Authors:** Xiaochen Zhu, Penny Miller-Ezzy, Tony Hall, Youhong Tang, Jianguang Qin, Yingying Zhao, Xiaoxu Li

**Affiliations:** 1 College of Science and Engineering, Flinders University, Adelaide, 5042 Australia; 2 Aquatic Science Centre, South Australian Research and Development Institute, Adelaide, 5024 Australia; 3 Mawson Analytical Spectrometry Services, Faculty of Sciences, Engineering and Technology, University of Adelaide, Adelaide, SA 5000 Australia; 4 College of Animal Science and Veterinary Medicine, Shenyang Agricultural University, Shenyang, 110866 China

**Keywords:** Fatty acid profile response, *Magallana gigas*, *Mytilus galloprovincialis*, Larval cryopreservation, Polyunsaturated fatty acids, Animal breeding, Developmental biology, Biological techniques, Non-model organisms

## Abstract

Larval cryopreservation techniques have been established in Pacific oysters and Mediterranean mussels. Although initial cryopreservation protocols for both species differed slightly in Ficoll PM 70 (FIC) concentration, better post-thaw larval survivability was produced in mussels than oysters. Furthermore, the post-thaw survivability in the latter could be improved significantly by the addition of lipids and antioxidants to the cryoprotectant agent. These findings have generated a unique opportunity to investigate the cryo-functions of both endogenous and exogenous fatty acids (FAs) in bivalves as mammalian studies indicated that lipid compositions could contribute the interspecific difference in gamete and embryo cryopreservation. Our study showed that the higher survivability of post-thaw larvae in mussels (65% vs. 34% in oysters) could be attributed to their higher proportion of total polyunsaturated fatty acids (PUFAs), especially C22:6, and higher resistance of FA profiles to cryopreservation. In oysters, on the other hand, their FA profiles were very sensitive to cryopreservation, with saturated FAs and PUFAs being significantly increased (from 37 to 41%) and decreased (from 35% to ~ 32%), respectively. Although exogenous lipids could significantly improve the survivability of post-thaw oyster larvae from 34 to 51%, their supplementation did not alter the response pattern of endogenous FA profiles to cryopreservation.

**Supplementary Information:**

The online version contains supplementary material available at 10.1038/s41598-024-76723-0.

## Introduction

Gamete and embryo/larva cryopreservation is an important approach to solving the problems of off-season seed supply, mating design, and unbalanced sex ratio of broodstock and asynchronous maturation in aquaculture breeding. Cryopreservation is crucial in germplasm conservation and genetic improvement programs^1,2^. As oocyte cryopreservation is more challenging than sperm due to its large size, complex structure, and high lipid content^3^, larval cryopreservation has become an alternative option to preserve the genetic materials of both parental origins.

In molluscs, Pacific oysters (*Magallana gigas*; formerly *Crassostrea gigas*) and Mediterranean mussels (*Mytilus galloprovincialis*) are economically significant species in global aquaculture, and the cryopreservation techniques in these two species have been established and are under gradual improvement over time^1,2,4–7^. Nevertheless, the performance of post-thaw larvae has been compromised due to cryopreservation, resulting in lower yields of D-stage larvae and spats than fresh controls^4,5,7^. The survivability of post-thaw larvae in Mediterranean mussels is better than in Pacific oysters after optimising the ratio of different components in the cryoprotectant agent (CPA)^4,5^. Recently, the survivability of post-thaw oyster larvae was further improved when the lipids and antioxidants were added to the CPA^7^.

Many factors, such as intracellular ice crystal formation, osmotic shock, overproduced reactive oxygen species (ROS), and lipid phase transition (LPT; a transition from a liquid phase to a crystalline-gel phase), can severely affect the survivability of post-thaw biological materials^3,8^. Among them, LPT at non-physiological temperatures is one of the key causes of cryoinjuries. When it occurs, the cytomembrane loses elasticity and permeability, resulting in the loss of cellular functionality^3,9^. Theoretically, gametes and embryos with lower LPT temperatures possess a better ability for cryoresistance^3,10^. LPT temperature is closely related to the composition of polyunsaturated fatty acids (PUFAs) because the kinks of double bonds hinder the tight packing of acyl chains and maintain cytomembrane fluidity^11^. For example, when compared with bovine embryos, domestic cat embryos have better post-thaw survivability due to the high abundance of unsaturated lipids^3,12^. Studies on aquatic sperm suggested that high levels of unsaturated fatty acid (UFA) and/or PUFA could contribute to the higher post-thaw sperm motility such as in the Atlantic salmon (*Salmo salar*)^13^ and the common carp (*Cyprinus carpio*)^14^.

In bivalve larvae, lipids serve as an essential energy source during the transition from lecithotrophy to exotrophy and the periods with low feeding activity, such as metamorphosis^15,16^. Some PUFAs also play a crucial role in larval cell structures, development and survival^17,18^. The decline in larval PUFA levels, such as arachidonic acid (ARA) and eicosapentaenoic acids (EPA) in blue mussels (*Mytilus edulis*), is positively related to larval mortality^19^. Cryopreservation can alter the fatty acid (FA) profiles of post-thaw sperm^20,21^, oocytes^22^, embryos^23^, and *Mytilus trossulus* larval cells^24^. Therefore, comparing the FA profiles and their response to larval cryopreservation between the larvae of Pacific oysters and Mediterranean mussels can enhance our understanding of factors contributing to the difference in post-thaw performance.

This study compares the FA profile responses to larval cryopreservation in Mediterranean mussels and Pacific oysters using two basal protocols for each species with slight differences in one CPA chemical only^4,5^. Since the survivability of post-thaw oyster larvae can be further improved by supplementation of 1-palmitoyl-2-oleoyl-sn-glycero-3-phosphocholine (POPC) and α-tocopherol (TOC) to CPA^7^, we also investigate the possible protective mechanisms of exogenous lipid and antioxidant. The study aims to (1) investigate the FA profile response of Mediterranean mussel and Pacific oyster larvae to cryopreservation, (2) determine the relationship between FA profile responses and post-thaw survivability, and (3) understand the roles of POPC and TOC for improving post-thaw oyster performance. This study could enhance our understanding of the roles of endogenous FAs and exogenous lipids and antioxidants in bivalve larval cryopreservation and offer a new approach to improve or develop cryopreservation techniques in aquatic species.

## Results

### Performance of post-thaw larvae

**Fig. 1:**
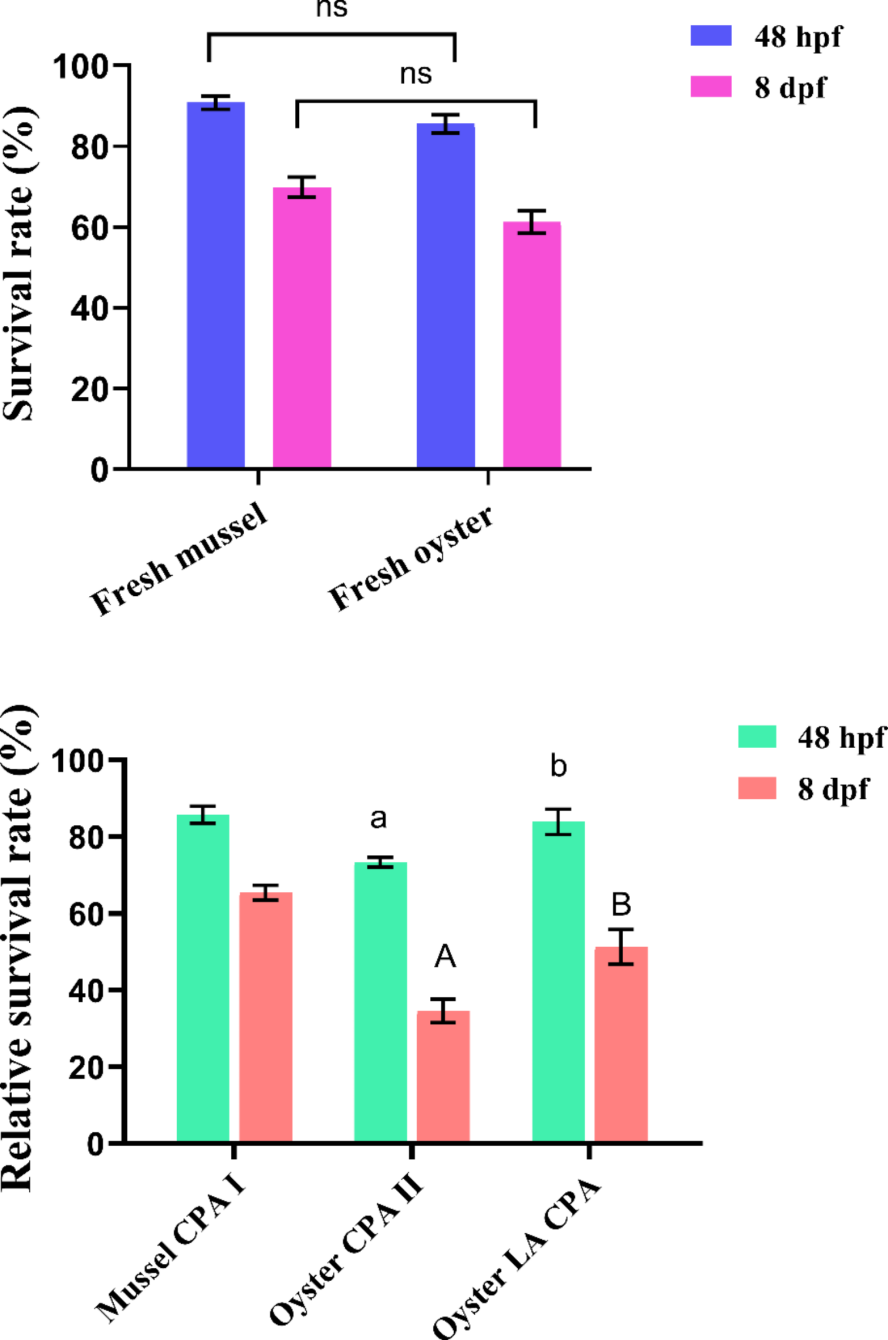
Survival rates of fresh controls (**A**) and relative survival rates of post-thaw larvae (**B**) at different ages in Mediterranean mussels and Pacific oysters (*n* = 3). ns indicates no significant difference between fresh oysters and mussels. Different lowercase or uppercase letters indicate significant differences between treatments within the same age in oysters.

At 48 h post fertilisation (hpf), survival rates of fresh control in Mediterranean mussels and Pacific oysters were 90.80 ± 1.63% and 85.53 ± 2.26% (Fig. 1A), respectively, which did not differ significantly. Figure. 1B shows that the relative post-thaw survival rate was 85.68 ± 1.54% in mussels cryopreserved with the Base CPA I. In oysters, the LA CPA treatment resulted in a higher relative survival rate (83.82 ± 1.58%) than the Base CPA II treatment (73.41 ± 1.69%).

At 8 d post fertilisation (dpf), survival rates of fresh control in mussels and oysters were 69.8 ± 2.50% and 61.2 ± 2.78% and did not differ significantly (Fig. 1A). The relative post-thaw survival rate was 65.52 ± 2.14% in mussels. Like at 48 hpf, the LA CPA treatment resulted in a higher relative survival rate (51.12 ± 2.42%) than the Base CPA II (34.4 ± 1.47%) in oysters (Fig. 1B).

## Responses of endogenous fatty acids to larval cryopreservation

The larval FA compositions before cryopreservation in Mediterranean mussels and Pacific oysters are shown in Table 2. Although fractions of total saturated fatty acids (SFAs) were similar: 37.34 ± 0.71% (mussels) vs. 37.62 ± 0.36% (oysters), significant differences were found in C12:0, C14:0, C15:0, C16:0, C20:0, and C22:0. Compared to mussels, the oyster controls had a significantly higher fraction of total monounsaturated fatty acids (MUFAs; *P* < 0.001): 27.04 ± 0.13% (oysters) vs. 20.54 ± 0.57% (mussels), which was mainly due to the higher percentage of C18:1 (13.61 ± 0.03%) in oysters than in mussels (4.66 ± 0.05%). The fraction of C20:1 in fresh mussel controls, on the other hand, was about 1.5% higher than in oysters. The total PUFAs also exhibited significant differences between the two fresh controls (*P* < 0.001), 42.11 ± 0.27% and 35.33 ± 0.28% in mussels and oysters, respectively. Specifically, fresh mussel larvae were rich in C22:6, C20:2, and C20:3, whereas oysters were abundant in C20:5 and C20:4.

No significant difference in FA profiles was observed between fresh and cryopreserved larvae in mussels (Supplementary Table 1.). In oysters, on the contrary, alterations were detected in many FAs between fresh and post-thaw larvae (Table 2). The post-thaw oyster larvae showed significant increases in total SFAs: 37.62 ± 0.36% (controls) vs. 40.80 ± 0.54% (LA CPA) and 40.62 ± 0.75% (Base CPA II) and decreases in total PUFAs: 35.33 ± 0.28% (controls) vs. 31.95 ± 0.54% (LA CPA) and 32.52 ± 1.01% (Base CPA II). More specifically, three PUFAs (C20:3, C20:4, and C22:6) were all reduced, whereas three SFAs were altered in different directions (increase in C14:0 and C16:0 but decrease in C22:0) in post-thaw oyster larvae.

In Pacific oysters, no significant difference in FA compositions was found between larvae cryopreserved by either CPA.

**Table 1:** Fatty acid composition (percentage of total FAs) of pre-cryopreserved larvae in Mediterranean mussels and Pacific oysters (*n* = 3). Asterisks indicate the significance of FAs composition in fresh controls between mussels and oysters. * *P* < 0.05; ** *P* < 0.01; *** *P* < 0.001.

Fatty Acid	Mussels	Oysters
**C8:0**	0.01 ± 0.01	0.00 ± 0.00
**C12:0**	0.03 ± 0.00^***^	0.01 ± 0.00
**C13:0**	0.01 ± 0.00	0.01 ± 0.00
**C14:0**	6.15 ± 0.06^***^	8.70 ± 0.05
**C15:0**	0.71 ± 0.04^**^	0.46 ± 0.01
**C16:0**	26.12 ± 0.6^*^	24.01 ± 0.4
**C17:0**	0.82 ± 0.05	0.71 ± 0.01
**C18:0**	3.32 ± 0.14	3.50 ± 0.06
**C20:0**	0.08 ± 0.01^**^	0.01 ± 0.00
**C21:0**	0.03 ± 0.02	0.14 ± 0.03
**C22:0**	0.02 ± 0.00^**^	0.05 ± 0.00
**C23:0**	0.01 ± 0.00	0.01 ± 0.00
**C24:0**	0.02 ± 0.01	0.02 ± 0.00
**∑ SFAs**	37.34 ± 0.71	37.62 ± 0.36
**C16:1**	9.65 ± 0.39	8.77 ± 0.10
**C18:1**	4.66 ± 0.05^***^	13.61 ± 0.03
**C20:1**	6.09 ± 0.23^**^	4.61 ± 0.01
**C22:1**	0.14 ± 0.03	0.05 ± 0.03
**∑ MUFAs**	20.54 ± 0.57^***^	27.04 ± 0.13
**C18:2**	5.03 ± 0.16	4.21 ± 0.08
**C18:3**	8.46 ± 0.20	8.23 ± 0.21
**C20:2**	3.66 ± 0.26^***^	1.22 ± 0.06
**C20:3w6c**	0.71 ± 0.01^***^	0.2 ± 0.01
**C20:4w6c**	0.33 ± 0.02^***^	3.7 ± 0.11
**C20:5w3**	4.57 ± 0.27^***^	11.14 ± 0.21
**C22:6w3c**	19.27 ± 0.17^***^	6.61 ± 0.24
**∑ PUFAs**	42.11 ± 0.27^***^	35.33 ± 0.28

**Table 2:** Fatty acid composition (percentage of total FAs) of pre- and post-cryopreserved larvae in Pacific oysters (n = 3). Different letters within each FA indicate significant difference between fresh and treated larvae in oysters.

Fatty acid	Fresh oysters	Base CPA II	LA CPA
C8:0	0.00 ±0.00	0.02 ±00.1	0.01 ±0.01
C12:0	0.01 ±0.00	0.01 ±0.00	0.01 ±0.00
C13:0	0.01 ±0.00	0.00 ±0.00	0.01±0.01
C14:0	8.70 ±0.05a	9.14 ±0.04b	9.14 ±0.04b
C15:0	0.46 ±0.01	0.44 ±0.00	0.45 ±0.00
C16:0	24.01 ±0.4a	26.51 ±0.64b	26.71 ±0.44b
C17:0	0.71 ±0.01	0.68 ±0.01	0.67 ±0.03
C18:0	3.50 ±0.06	3.59 ±0.10	3.63 ±0.08
C20:0	0.01 ±0.00	0.01 ±0.00	0.02 ±0.00
C21:0	0.14 ±0.03	0.16 ±0.02	0.19 ±0.05
C22:0	0.05 ±0.00a	0.04 ±0.00b	0.03 ±0.00b
C23:0	0.01 ±0.00	0.00 ±0.00	0.01 ±0.00
C24:0	0.02 ± 0.00	0.01 ± 0.00	0.01 ± 0.01
Σ SFA	37.62 ± 0.36a	40.62 ± 0.75b	40.80 ± 0.54b
C16:1	8.77 ± 0.10	8.72 ± 0.07	8.72 ± 0.13
C18:1	13.61 ± 0.03	13.41 ± 0.11	13.42 ± 0.12
C20:1	4.61 ± 0.01	4.56 ± 0.26	4.56 ± 0.26
C22:1	0.05 ± 0.03	0.15 ±0.04	0.19 ±0.07
C24:1	0.00 ± 0.00	0.01 ±0.01	0.00 ±0.00
Σ MUFA	27.04 ± 0.13	26.85 ±0.28	27.25 ±0.06
C18:2 total	4.21 ± 0.08	4.06 ±0.14	4.05 ±0.10
C18:3 total	8.23 ± 0.21	7.59 ±0.38	7.31 ±0.17
C20:2 total	1.22 ±0.06	1.48 ±0.12	1.50 ±0.05
C20:3w6c	0.2 ±0.01a	0.03 ±0.03b	0.02 ±0.02b
C20:4w6cAA	3.7 ±0.11a	3.12 ±0.12b	3.21 ±0.08b
C20:5w3EPA	11.14 ±0.21	10.43 ±0.32	10.35 ±0.09
C22:6w3cDHA	6.61 ±0.24a	5.81 ± 0.37ab	5.50 ±0.24b
Σ PUFA	35.33 ±0.28a	32.52 ±1.01b	31.95 ±0.54b

## Discussion

Investigations of FA profiles have been done in bivalve oocytes or larvae^17,18,25,26^. However, those studies have primarily focused on examining the effects of diet on larval performance or assessing the correlation between FA profiles of broodstock and the quality of resultant gametes or larvae. For example, Adams et al. examined the effect of broodstock nutrition on the FA profile of Pacific oyster oocytes^27^. To our knowledge, this study was the first to investigate the response of larval FA profiles to cryopreservation in molluscan species.

In this study, post-thaw larval survival rates at either 48 hpf or 8 dpf were similar to the literature using the same CPAs and protocols in Mediterranean mussels and Pacific oysters^4,5,7^, indicating the reproducibility of the published larval cryopreservation techniques in these species. These two ages were also critical for assessing their performance, as previous studies have shown that the growth and relative survival rates of post-thaw larvae are similar to the controls from 8 dpf in both species^4–7^. In addition, the Mediterranean mussel larvae exhibited better cryotolerance than Pacific oysters. In this study, the relative post-thaw survival rates were 65.43 ± 1.93% at 8 dpf in mussels, whereas only 51.31 ± 4.51% (LA CPA) and 34.53 ± 3.05% (Base CPA II) in oysters.

Mammalian cryopreservation studies have shown that the species-specific proportion of unsaturated lipids in gametes or embryos is closely related to their differences in cryotolerance between species. The comprehensive review by Amstislavsky et al.^3^ indicated embryos and oocytes with abundant unsaturated lipids [e.g., mouse and rat^28,29^) exhibit higher birth rates after cryopreservation than those rich in saturated lipids [e.g., ovine and bovine^30,31^). Studies in fish sperm also indicated a tendency that sperm with high PUFA and low SFA levels could result in better fresh or post-thaw motility and fertility^13,14,32^. PUFAs usually exhibit lower LPT temperatures due to their abundance in double carbon bonds, which can create kinks to reduce the packing of acyl chains at low temperatures^33^. Therefore, a high proportion of PUFAs could help maintain cytomembrane fluidity at a low temperature and thus reduce the damages caused by the osmotic shock and ice crystal formation during cryopreservation^3^. In this study, the proportion of total PUFA in mussel controls (42.11 ± 0.27%) was significantly higher than that in the oyster controls (35.33 ± 0.28%), which could contribute to the higher post-thaw larval survival rates in mussels than in oysters and also support the findings in cryopreservation studies in mammalian oocytes and embryos and fish sperm.

Among PUFAs, the C22:6 fraction has shown positive correlation with better post-thaw sperm survivability in humans and mammalians^34,35^ and involved in maintaining membrane structures and functions during embryogenesis in bivalves^18,36^. Therefore, the dominance of C22:6 (19.27 ± 0.17%) found in the mussel PUFAs in this study could be a specific PUFA factor contributing to their higher post-thaw larval survival rate than oysters. However, the dominance of C20:5 in fresh oyster larvae (11.14 ± 0.21%) might not be able to improve the cryotolerance, as this PUFA mainly functions as an energy source and a precursor of eicosanoids^18,36,37^. Although bivalves usually have insufficient capability to elongate and desaturate short-chain saturated FAs to long-chain PUFAs^38–40^, the lipid profiles of their gametes and the embryos or larvae produced by these gametes could be altered through the broodstock diet manipulation during gametogenesis^17,27^. For example, the C22:6% in oocytes is increased by feeding Pacific oyster broodstock with a diet rich in C22:6 ^27^. Therefore, oocyte lipid modification may provide a new strategy to improve the larval cryopreservation in bivalves. In South Australia, mussels and oysters mature and spawn over cold and warm seasons, respectively. According to Liu et al.^41^, the water nutrient profiles over these periods also differ, which could affect the nutrient intake of the broodstock used in this study. This could, in turn, contribute to the difference in the larval FA profile and cryo-sensitivity found between these two species.

In this study, the larval FA profile was not affected by the cryopreservation in mussels. In oysters, on the other hand, it was changed in larvae cryopreserved with either CPA, resulting in a decrease in total PUFAs and an increase in total SFAs. These and the post-thaw survival results indicate that the cryopreservation protocols used in this study have less impact on larvae in Mediterranean mussels than Pacific oysters. The changes in proportion between PUFAs and SFAs after cryopreservation have been widely reported in mammalian and aquatic species^21,24,42–44^. Lipid peroxidation induced by ROS during cryopreservation is one of the reasons for these changes^20,42^. Our results in mussels are supported by the recent investigation on the redox status of post-thaw larvae in this species, where no significant differences were observed in ROS production and total antioxidant capacity^45^. However, further study on the reasons causing FA profile changes in Pacific oysters is required as cryopreservation did not change ROS production in post-thaw larvae, despite a reported increase in total antioxidant capacity in another study^46^.

In Pacific oysters, post-thaw survival rates of the larvae cryopreserved with the LA CPA were higher than those with the Base CPA II at both 48 hpf and 8 dpf, which are in line with a previous study by Zhu et al.^7^. However, no significant differences in FA profiles were observed. Therefore, the protective function of POPC and TOC during cryopreservation was not through the maintenance of FA profiles in oysters. Further investigations on the interaction between larval cell membrane and POPC and TOC, and their impacts on redox status, DNA integrity and gene expression in the post-thaw larvae may provide insight into their cryo-protective function in bivalve species.

## Conclusions

The higher fraction of PUFAs, especially C22:6, in fresh mussel larvae might contribute to their better post-thaw survivability than oysters. The larval FA profiles also showed higher cryo-resistance in Mediterranean mussels than in Pacific oysters, as they remained unaltered after cryopreservation in the former and shifted with increased SFAs and decreased PUFAs in the latter. Although exogenous POPC and TOC supplementation has significantly improved the post-thaw larval survivability in Pacific oysters, it did not change the responding pattern of endogenous FA profiles in this species.

## Materials and methods

### Larval preparation

#### Mediterranean mussels

Broodstock mussels were provided by Eyre Peninsula Seafood, South Australia (SA) during their natural spawning season in 2022 (mid-June to mid-August in SA). Mussels were induced to spawn individually through thermal shock as described by Liu & Li^47^ in 1 μm filtered saltwater (FSW). Gamete quality was assessed under a light microscope. Males with vigorous motility sperm (> 90%) and females with round shape and homogenous cytoplasm oocytes were selected. Oocytes were collected on a 35 μm sieve, with an 80 μm sieve on top to remove the large debris. Sperm was filtered through a 25 μm sieve. Sperm from at least five males and oocytes from at least eight females were pooled in each batch. A sperm-to-oocyte ratio of 20:1 was used for 10 min fertilisation. The fertilised eggs were washed and incubated at 20 ℃ at a stocking density of approximately 15 eggs/mL. Healthy trochophore larvae were collected from the top layer of the tank at 24 hpf. The larvae collected in each tank were transferred into a 10 mL Falcon tube, adjusted to 4 × 10^5^ larvae/mL by adding FSW, and stored on ice for < 15 min before the experiment.

## Pacific oysters

The larval preparation method described by Liu et al.^4^ was used in this study. During the natural spawning season of Pacific oysters in SA (December of 2022 and January of 2023), broodstock sourced from local growers was assessed visually, and those with clear gonad spawning lines were stripped. The gametes were then examined under a light microscope. Females with mature oocytes and males with > 95% active sperm were used in the experiments. Gametes were collected individually. After remaining undisturbed in 1 μm FSW for 2 h, oocytes were collected on a 20 μm screen with a 75 μm screen on the top to remove debris. Sperm suspensions were filtered through a 25 μm screen to remove gonad debris. Sperm from at least four males and oocytes from at least five females were pooled in each batch. A sperm-to-oocyte ratio of 20:1 was used for 10 min fertilisation. The fertilised eggs were then washed and incubated at 21 ℃ at a stocking density of approximately 15 eggs/mL. Healthy trochophore larvae were collected from the top layer of the tank at 14 hpf. The larvae collected from each tank were transferred into a 10 mL Falcon tube, adjusted to 4 × 10^5^ larvae/mL by adding FSW and stored on ice for < 15 min before being used in experiments.

### Cryoprotectant agent preparation

The CPA described by Liu et al.^5^ was used for mussel larval cryopreservation in this study (Base CPA I), which consists of 10% (v/v) ethylene glycol (EG), 7.5% (w/v) Ficoll PM 70 (FIC), and 0.2% (w/v) polyvinylpyrrolidone (PVP). Two CPAs were employed for oyster larval cryopreservation: (1) Base CPA II, comprising 10% (v/v) EG, 5% (w/v) FIC, and 0.2% (w/v) PVP^4^; and (2) LA CPA, consisting of Base CPA II plus 0.25 mg/mL POPC and 1 mg/mL TOC^7^. Due to the hydrophobicity of POPC and TOC, they were emulsified into a liposome solution in the Base CPA II following the method described by Zhu et al.^7^. Briefly, POPC and TOC were initially dissolved in chloroform and ethanol at 0.25 mg/µL, respectively. After the required volume of solution (predetermined quantity of POPC or TOC) was transferred into a 2 mL vial, ethanol or chloroform was purged with inert N_2_ gas. Then the vial was added with required volume of Base CPA II and subjected to high-speed sonication (Soniclean, Thebarton, Australia) for 30 min to 1 h.

In this study, all CPAs were prepared twice the required concentration for the experiment. The CPA concentration required for the experiment was achieved when equal volumes of CPA solution and larval suspension were mixed.

### Freezing and thawing protocol

The freezing and thawing protocols developed by Liu et al.^4,5^ were identical in both species and used in this study. The CPA and larval suspension were mixed on ice for 10 min before being transferred into 0.5 mL straws (IMV, France). They were then loaded into a programmable controller (CL-3300; CryoLogic, Mulgrave, Australia) and maintained at 0 °C for 5 min. They were frozen at − 1 °C/min from 0 to − 10 °C followed by − 0.3 °C/min from − 10 °C to − 34 °C before being plunged into liquid nitrogen (LN).

After at least two months of storage in the LN, samples were thawed in a 28 °C water bath and then transferred to a 21 °C water bath for recovery. The larvae from each straw were expelled into a 5 mL vial and mixed with 0.5 mL FSW (including 9% sucrose) for 10 min at 21 °C. A volume of FSW equal to that in the vial was added twice at 10 min intervals to reduce the intracellular CPA concentration.

### Cryopreservation experiments

#### Mediterranean mussels

In Mediterranean mussels, six cryopreservation cycles were conducted with different batches of broodstock. In each cycle, 23 straws (the maximum capacity of the cryochamber) were cryopreserved. A subsample of fresh larvae was also collected from each batch and plunged into LN as a control. Before lipid extraction, control samples from two consecutive cycles were pooled as one fresh control replicate (three replicates in total).

After post-thaw recovery, the mussel larvae from each cycle were collected on a 35 μm sieve, transferred into a 5 mL cryotube and plunged into LN. Before lipid extraction, larvae from two consecutive cryopreservation cycles were pooled as one replicate (three replicates in total).

### Pacific oysters

Pacific oyster larvae were cryopreserved by nine cycles with different batches of broodstock. Eleven straws were cryopreserved within each cycle using Base CPA II and LA CPA, respectively. A control sample was also established in the same way as in mussels.

The preparation of treated samples was similar to that described for mussels. To remove the residual POPC and TOC, the post-thaw LA CPA larvae were rinsed with FSW for approximately 10 min on a 20 μm sieve before being stored in LN.

Before lipid extraction, samples from three consecutive cycles were pooled into one fresh control, Base CPA II treatment and LA CPA treatment, respectively, resulting in three replicates for each treatment.

### Performance of post-thaw larvae

At the second, fourth and sixth cryopreservation cycles in the Mediterranean mussel experiment and the third, sixth and ninth cycles in the Pacific oyster experiment, a subsample of fresh larvae similar to the amount of one 0.5 mL straw was used as the fresh control for larval performance assessment. One straw in each treatment was thawed after 12 h in LN. Their survival rates of D-stage larvae were assessed at 48 hpf. D-stage larvae from each replicate were collected using a 25 μm and 50 μm screen for oyster and mussel larvae, respectively. The larvae were then resuspended to 50 mL with FSW. After being mixed thoroughly, five subsamples (50 µL each) were taken from each replicate using a micropipette. Then 10 µL of Lugol’s solution was added to each subsample to fix the larvae. The number of normal D-stage larvae was counted under a microscope. The larval number in each replicate was calculated by multiplying the average count of five subsamples by 1000. The survival rate of D-stage larvae in each replicate was determined by dividing the number of D-stage larvae produced by the number of larvae initially used at the beginning of the treatment. The relative survival rates were calculated by dividing the survival rate of each replicate by the survival rate of the fresh control from the same batch to compare the post-thaw survivability between species.

As the performance of post-thaw larvae in these two species tends to stabilise after 8 dpf^4,5,7^, their survival rates were also measured at 8 dpf in this study. After the first assessment at D-stage, the remaining larvae were cultured until 8 dpf. The procedures described by Wang et al.^48^ and Li^49^ for Mediterranean mussel and Pacific oyster larval cultivation, respectively, were followed. The method used to measure the relative survival rates at 8 dpf was the same as that used for D-stage larvae.

### Total lipid extraction and fatty acid profile analysis

Total lipids were extracted from freeze-dried larvae using Folch’s method^50^. The lipid extracts were then hydrolysed to yield free FAs, which were subsequently transformed into methyl esters and analysed using gas chromatography-mass spectrometry (GC-MS; Agilent 5977/7890) operating in SIM/Scan mode with data processing conducted using ChemStation software. Identification was by comparison to a Supelco 37 component fatty acid methyl esters (FAME) mix (Supelco CRM47885) with methyl nonadecanoate internal standard (Supelco 74208) and quantification was conducted using the selective ion monitoring (SIM) data. In this study, total lipid extraction and transfesterification were performed by the South Australia Research and Development Institute (SARDI) Environment and Analytical Laboratory. GC-MS was conducted at Mawson Analytical Spectrometry Services, the University of Adelaide.

### Statistical analysis

Statistical analyses were performed using SPSS 20.0 software. Data are presented as mean ± standard error (SE). An independent-sample T-test was used to assess the survival rates in fresh controls between mussels and oysters at 48 hpf or 8 dpf, each FA composition between mussels and oysters in fresh controls and between fresh and post-thaw larvae in mussels. One-way analysis of variance (ANOVA) with the Waller-Duncan test was applied to compare the relative larval survival rates among post-thaw mussel and oyster larvae and each FA composition between fresh and post-thaw larvae in Pacific oysters. The significance level was set at *P* < 0.05.

## Electronic supplementary material

Below is the link to the electronic supplementary material.

Supplementary Material 1

## Data Availability

The datasets used and/or analysed during the current study available from the corresponding author on reasonable request.

## Abbreviations

CPA: Cryoprotectant agent
EG: Ethylene glycol
FIC: Ficoll^®^ PM 70
PVP: Polyvinylpyrrolidone
POPC: 1-palmitoyl-2-oleoyl-sn-glycero-3-phosphocholine
TOC: α-tocopherol
PUFAs: Polyunsaturated fatty acids
MUFAs: Monounsaturated fatty acids
FAs: Fatty acids
LPT: Lipid phase transition
ROS: Reactive oxygen species
LN: Liquid nitrogen
FWS: Filtered saltwater
