# Supplementary material for: Different responses of larval fatty acid profiles to cryopreservation in two commercially important bivalves
Source: Sci Rep. 2024 Oct 19;14:24582. doi: 10.1038/s41598-024-76723-0 (PMC11490629; doi:10.1038/s41598-024-76723-0)
Supplement: Supplementary file 1 — Supplementary Material 1 [file 41598_2024_76723_MOESM1_ESM.docx]

Supplementary table 1. Fatty acid composition (percentage of total FAs) of pre- and post-cryopreserved larvae in Mediterranean mussels (n = 3)
